# Development of a novel lncRNA-derived immune gene score using machine learning-based ensembles for predicting the survival of HCC

**DOI:** 10.1007/s00432-024-05608-6

**Published:** 2024-02-09

**Authors:** Qun Cai, Guoqi Li, Mingyan Zhu, Tingting Zhuo, Jiaying Xiao

**Affiliations:** 1 Department of Infectious Diseases and Liver Diseases, Ningbo Medical Center Lihuili Hospital, Affiliated Lihuili Hospital of Ningbo University, 1111 Jiangnan Rd., Ningbo, 315100 China; 2 College of Bioinformatics Science and Technology, Harbin Medical University, Harbin, 150036 Heilongjiang China

**Keywords:** LncRNAs, HCC, EREG, Immune gene, Biomarker

## Abstract

**Background:**

Long noncoding RNAs (lncRNAs) are implicated in the tumor immunology of hepatocellular carcinoma (HCC).

**Methods:**

HCC mRNA and lncRNA expression profiles were used to extract immune-related genes with the ImmPort database, and immune-related lncRNAs with the ImmLnc algorithm. The MOVICS package was used to cluster immune-related mRNA, immune-related lncRNA, gene mutation and methylation data on HCC from the TCGA. GEO and ICGC datasets were used to validate the model. Data from single-cell sequencing was used to determine the expression of genes from the model in various immune cell types.

**Results:**

With this model, the area under the curve (AUC) for 1-, 3- and 5-year survival of HCC patients was 0.862, 0.869 and 0.912, respectively. Single-cell sequencing showed EREG was significantly expressed in a variety of immune cell types. Knockdown of the EREG target gene resulted in significant anti-apoptosis, pro-proliferation and pro-migration effects in HepG2 and HUH7 cells. Moreover, serum and liver tissue EREG levels in HCC patients were significantly higher than those of healthy control patients.

**Conclusion:**

We built a prognostic model with good accuracy for predicting HCC patient survival. EREG is a potential immunotherapeutic target and a promising prognostic biomarker.

**Supplementary Information:**

The online version contains supplementary material available at 10.1007/s00432-024-05608-6.

## Introduction

Hepatocellular carcinoma (HCC) has major impacts on the world health burden due to its high mortality rate and poor prognosis. This is largely attributable to advanced disease progression at diagnosis and ineffective treatments (Bray et al. 2018; Sayiner et al. 2019). Therefore, early diagnosis and intervention of "high-risk" HCC are essential for improving clinical outcomes (Foerster et al. 2022). The liver is considered an immune organ, with the host immune system playing an important role in recognizing and targeting tumor cells in immunotherapy (Hafezi et al. 2021). Therefore, it is important to identify HCC-specific immunodiagnostic biomarkers and to explore the pathological mechanisms underlying HCC, especially in relation to immunotherapy targets. Although immune checkpoint inhibitors (ICIs) recently became a revolutionary new modality in cancer immunotherapy (Cheng et al. 2020), relatively few patients benefit from ICI therapy (Dong et al. 2021). Reliable biomarkers for the optimization of HCC drug therapy and prognosis are urgently needed in this new era of individualized therapy.

Long non-coding RNAs (lncRNAs) of > 200 nucleotide length have been implicated in the pathogenesis of various cancer types, including HCC, by regulating the biological behavior of tumor cells (Li et al. 2020). LncRNAs participate in the hallmarks of cancer (e.g., sustained proliferation, apoptosis escape, enhanced angiogenesis, and increased invasive ability) by binding to DNA, RNA or protein, or by encoding small peptides (Chang et al. 2016; Huang et al. 2020; Zuo et al. 2020). The role of most lncRNAs in HCC are currently unknown, but their unique properties suggest they may be useful in the diagnosis and treatment of HCC (Wang et al. 2021a). LncRNAs play key roles in regulating the liver microenvironment and chronic liver disease, including the regulation of immune responses, liver regeneration and oxidative stress (Huang et al. 2020; Wei et al. 2019). Dysregulation of lncRNAs can therefore lead to chronic hepatitis, liver regeneration and oxidative stress, resulting in the development of HCC.

A better understanding of lncRNA-related immune genes should therefore provide new insights into the pathogenesis of HCC. Here, we constructed and validated a lncRNA-related, immune gene-based prognostic score for the prediction of HCC patient outcome. Further in vivo and in vitro work is required to understand the pathogenesis of immune-related related genes in HCC. This should ultimately lead to new tools for early diagnosis and treatment of HCC.

## Methods

### Data acquisition and processing

Data on HCC (TCGA-LIHC) was obtained from the UCSC xena database and included results for gene methylation, gene mutation, clinical parameters, patient survival, and copy number variation. For expression profile data, gene types were annotated according to "gencode.v22.annotation.gtf" from the Genecode database, genes for "protein_coding" were annotated as mRNAs, and genes for "gencode.v22.long_noncoding_RNAs.gtf" were annotated as lncRNAs. The "normalizeGeneCounts" function from the "GenomicFeatures" package was applied to convert raw count data to TPM data. For methylation data, only methylation sites with a univariate Cox regression* p* < 0.05 were retained. For mutation data, a mutated gene (regardless of the type of mutation) was defined as 1, while non-mutated genes (wildtype) were defined as 0. Only the genes mutated in > 10% of samples were retained. Expression profiles and survival information were extracted from the ICGC-JP cohort in the ICGC database (n = 232), while GSE76427 expression profiles and survival information were obtained from the GEO database (n = 95). Only cases with a survival time > 30 days and with corresponding multi-omics data were retained from TCGA-LIHC (n = 261). In total, 2483 immune-related genes from 17 immune-related pathways were analyzed in the ImmPort database.

### Identification of sample subtypes

The tumor purity of TCGA-LIHC samples was determined using the ESTIMATE algorithm. The partial correlation coefficient between mRNA and lncRNA was then calculated after removing the influence of tumor purity on the correlation analysis. Finally, immune-related lncRNAs were extracted from the "ImmLnc package". mRNAs that were common to the three cohorts (TCGA-LIHC, ICGC-JP and GSE76427) were intersected with 2483 immune-related genes to obtain immune-related mRNAs. Cluster analysis was then performed on the mRNA, lncRNA, gene methylation and gene mutation data by integrating 10 algorithms (iClusterBayes, SNF, IntNMF, CIMLR, MoCluster, PINSPlus, NEMO, COCA, LRAcluster Consensus Clustering) in the MOVICS package (Lu et al. 2021). From this analysis we obtained two subtypes (CS1 and CS2) and selected the top 100 genes with significant differential expression between them in order to build a prognostic model.

### Construction of the prognostic model

To identify prognosis-related genes, Cox univariate regression analysis was performed on 100 genes in the CS1 and CS2 subgroups from TCGA-LIHC. A risk score was obtained using the following formula: ($ICI.score(Risk score))$= $\sum {\beta PC1}_{A}$—$\sum {\beta PC1}_{B}$) (Zhang et al. 2020), where A is CS1 and B is CS2. Samples from each dataset were separated into high- or low-score groups based on the median score. The TCGA database was used for the training set, while the ICGC and GEO databases were used for the validation sets. Characteristics with significant prognostic value were identified after further integration of the clinical characteristics with risk scores in the TCGA-LIHC database using univariate and multivariate Cox analyses. This also allowed construction of the nomograms.

### Comparison of the high- and low-score groups

TCGA samples were classified as high- or low-score according to the median score. The three characteristics of metabolism, tumor microenvironment and tumor features were identified from the “IOBR package”. The “calculate_sig_score” function was used to calculate the score for each feature, and “maftools package” was used to visualize mutation signatures for high- and low-score groups. Quantification of immune cell infiltration was performed with “CIBERSORT, EPIC, xCell, TIMER, quantIseq, and MCPcounter” using IOBR package. The infiltration of 28 immune cell types was quantified using the ssGSEA method. The sensitivity of high- and low-score groups to 138 drugs was predicted with “pRRophetic package”. At a *p*-value setting of 0.000001, 27 drugs showed a significant difference in sensitivity between the two groups.

### Single-cell sequencing

The GSE125449 dataset (composed of Set-1 and Set-2) was used to assess correlations between mRNA expression and immune cell types in the model. The “Seurat package” (Butler et al. 2018) was first used to process the data, followed by the “loomR package” to convert the seurat objects into loom files. These were converted to h5ad files using the “scanpy package” in python, which were then uploaded to the cellTypist website for cell type annotation. “Immune_All_Low.pkl” was used as the reference dataset.

### Cell cultures and transfection

Human HCC cell lines (HepG2, HUH7) were obtained from the Chinese Academy of Science (Shanghai, China). To generate HCC cells with knockdown of EREG (HepG2 KD and HUH7 KD, respectively), the cells were transfected (Lipofectamine 2000, Invitrogen, USA) with 50 nM of specific siRNA construct (Gene-Pharma, China) for the human EREG sequence. The negative control (NC) for these experiments was a scrambled siRNA construct. Cells were grown at 37 °C and 5% CO_2_ in Dulbecco’s modified eagle medium containing 10% fetal bovine serum and transfected once the confluency reached 50%. Specific siRNA constructs for EREG are shown in Supplementary Table 1.

### In vivo and in vitro experiments to validate EREG

The expression level of EREG was evaluated in serum by ELISA (Human epiregulin [EREG] ELISA kit, CSB-EL007779HU) and in liver tissue by immunohistochemistry (Epiregulin Polyclonal Antibody, PA5-46,969) in 50 HCC patients and 50 healthy controls. Comprehensive functional analysis for cell migration, cell cycle and cell proliferation was performed following knockdown of EREG in HepG2 and HUH7 cells for 24 h. Cells were then evaluated for apoptosis (Annexin V-FITC, C1062L), for the cell cycle by flow cytometry (cell cycle detection kit, KGA512), and for proliferation using CCK-8 (cell counting kit-8, K1018).

### Statistical analysis

R software (Version 4.0.3) and SPSS 21.0 (SPSS, Inc, Chicago) were used for the data analysis. In vitro assays were performed independently and at least three times each. Normally distributed continuous variables are presented as the mean ± standard deviation, while non-normally distributed variables are shown as the median with inter-quartile range (P_25_-P_75_). The Student’s t-test was used to perform pairwise comparison of normally distributed variables, and the Wilcoxon test for non-normally distributed variables. Statistical significance was set at *P* < 0.05.

### Data availability

Datasets from this study are deposited in online repositories. The names and accession number(s) for these datasets are listed in the article and Supplementary Material.

## Results

### Construction of the prognostic model and evaluation of its accuracy

**Fig. 1:**
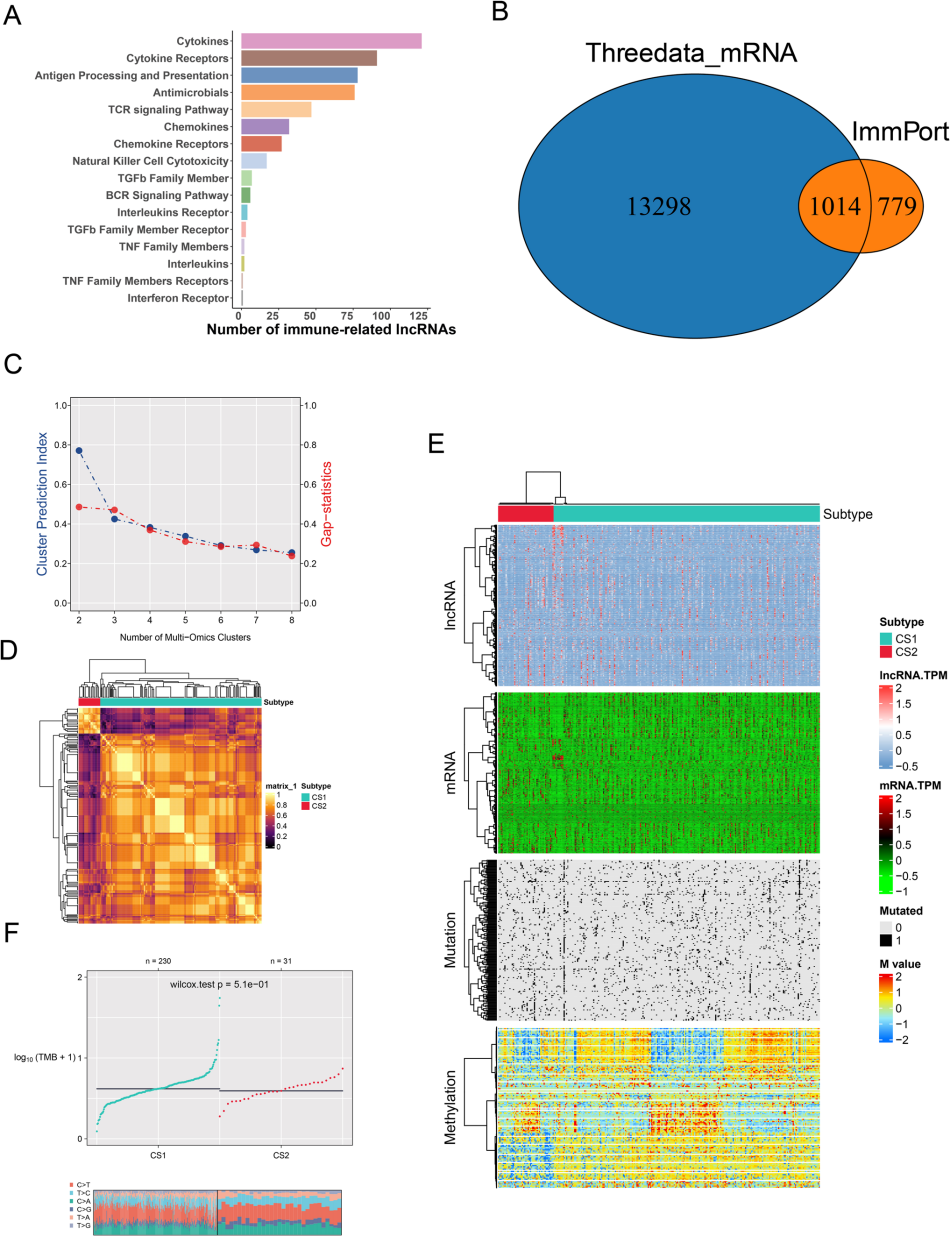
Analysis of immune genes associated with IncRNA. **A** The signaling pathways in which lncRNAs are mainly involved. **B** Intersection Venn diagram of mRNA from the three data sets (TCGA, GEO and ICGC) and immune-related genes from the ImmPort database. **C** TCGA-LIHC multi-omics data clustering coefficient determination. **D** Consensus heatmap based on multiple multi-omics integrative clustering algorithms. **E** TCGA-LIHC multi-omics comprehensive clustering heatmap based on sample dendrogram. **F** Differences in tumor mutational burden (TMB) between the two subtypes CS1 and CS2

In all, 800 immune-related lncRNAs were found in the TCGA-LIHC using the ImmLnc algorithm. These lncRNAs are involved mainly in signaling pathways (Fig. 1A). mRNAs in the three datasets were intersected with immune-related genes from the ImmPort database, and 1014 immune-related genes were finally included (Fig. 1B). Cluster analysis was performed based on 800 lncRNAs, 1014 mRNAs, gene mutations and gene methylation, finally giving rise to two subtypes referred to here as CS1 and CS2 (Fig. 1C, D). Figure 1E shows the heatmap highlighting the differences between the two subtypes. Significant differences in gene mutations between them were also found in TMB (Fig. 1F).

**Fig. 2:**
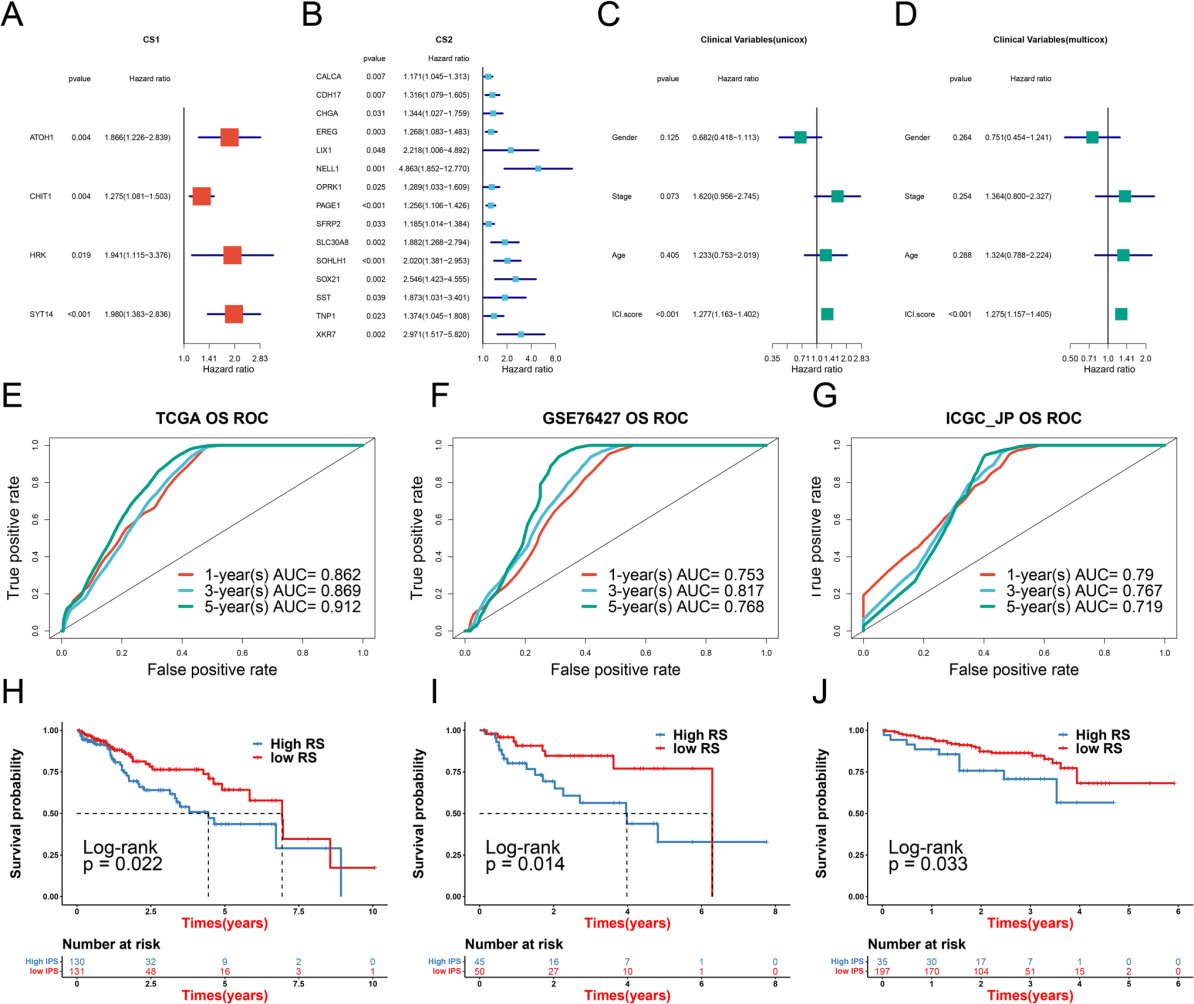
Construction of a prognostic model and evaluation of its efficacy. **A** Four significantly different genes were associated with prognosis in CS1. **B** Fifteen significantly different genes were associated with prognosis in CS2. **C** Univariate and **D** multivariate Cox regression analyses were used to determine the correlation between risk score, clinical variables and prognosis. **E** ROC analysis of 1-, 3- and 5-year overall survival in the TCGA cohort used as the training set. ROC analysis of 1-, 3-, and 5-year overall survival in the GEO **F** and ICGC **G** cohorts used as validation sets. **H** Overall survival analysis of high- and low-score groups in the training TCGA cohort. Overall survival analysis of high- and low-score groups in the validation GEO **I** and ICGC **J** cohorts

Four of the top 100 significantly different genes were associated with prognosis in CS1 (Fig. 2A), while 15 were associated with prognosis in CS2 (Fig. 2B). Univariate and multivariate Cox regression analysis was used to integrate risk scores with clinical variables. This revealed that only the ICI.score (Riskscore) had significant prognotic value (Fig. 2C, D). The prognostic model based on these genes had good performance in both the training and validation sets. The area under the ROC curve for 1-, 3- and 5-year survival of HCC patients in both training sets were all greater than 0.719, with the highest being 0.912 (Fig. 2E–G). The median value was used to define low- and high-score groups. Patients in the high-score group showed significantly better prognosis than those in the low-score group (Fig. 2H-J). We further constructed a nomogram using risk scores to predict HCC patient outcome and validated this using a calibration curve (Supplementary Fig. 1).

### Comparison of characteristics for high- and low-score groups

**Fig. 3:**
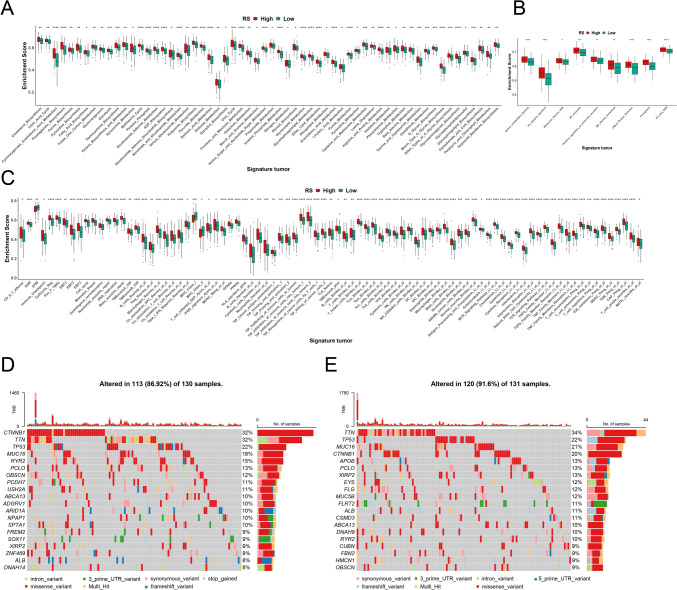
Comparison of the characteristics between high- and low-score groups. Significant differences in metabolism **A**, tumor microenvironment **B**, and tumor **C** characteristics were observed between the two groups. **D** Gene mutation map of the high-score group, and **E** of the low-score group. *p < 0.05; **p < 0.01; ***p < 0.001; ****p < 0.0001

**Fig. 4:**
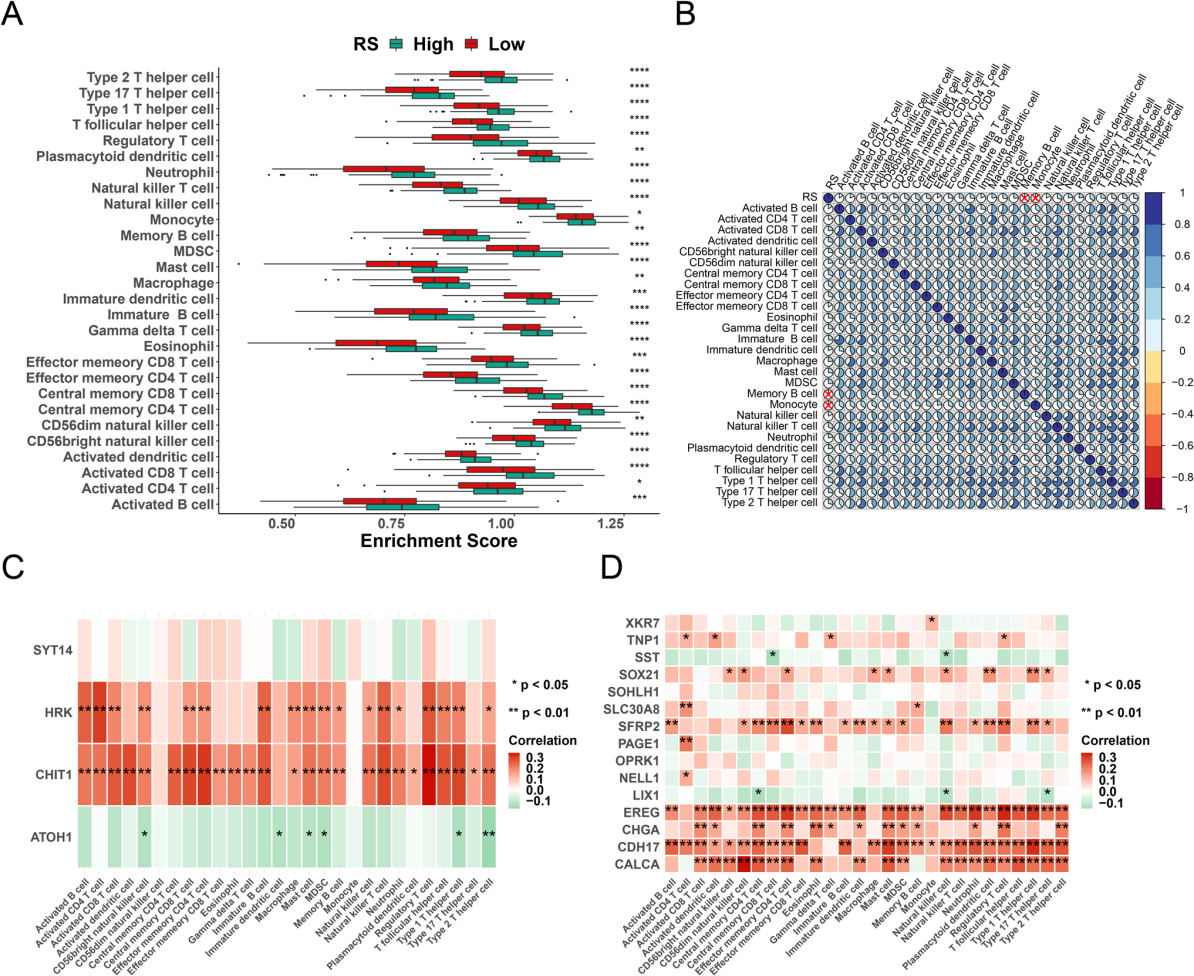
**A** Box plot of differences in the content of 22 types of immune cell between the high- and low- score groups. **B** Pearson correlation heatmap of 22 types of immune cell in the TCGA-LIHC. **C** Spearman correlation heatmap of four genes from the CS1 subtype and 22 types of immune cell. **D** Spearman correlation heatmap of 15 genes from the CS2 subtype and 22 types of immune cell

High- and low-score groups were systematically compared. Significant differences were found in metabolism, tumor microenvironment, and tumor properties (Fig. 3A–C). Comparison of the gene mutation profile also revealed significant differences between the two groups. Gene mutation maps for the high- and low-score groups are shown in Figs. 3D, E, respectively. Differences in 28 immune cell types are also shown in Figs. 4A, B, while genes used to build the prognostic model and their correlation with immune cells are shown in Figs. 4C, D. Clear differences in sensitivity (IC50) to antitumor drugs were found between high- and low-score groups. The top 27 drugs showing significant differences in sensitivity are listed in Supplementary Fig. 2.

### Differential expression of prognosis-related genes in immune cells

**Fig. 5:**
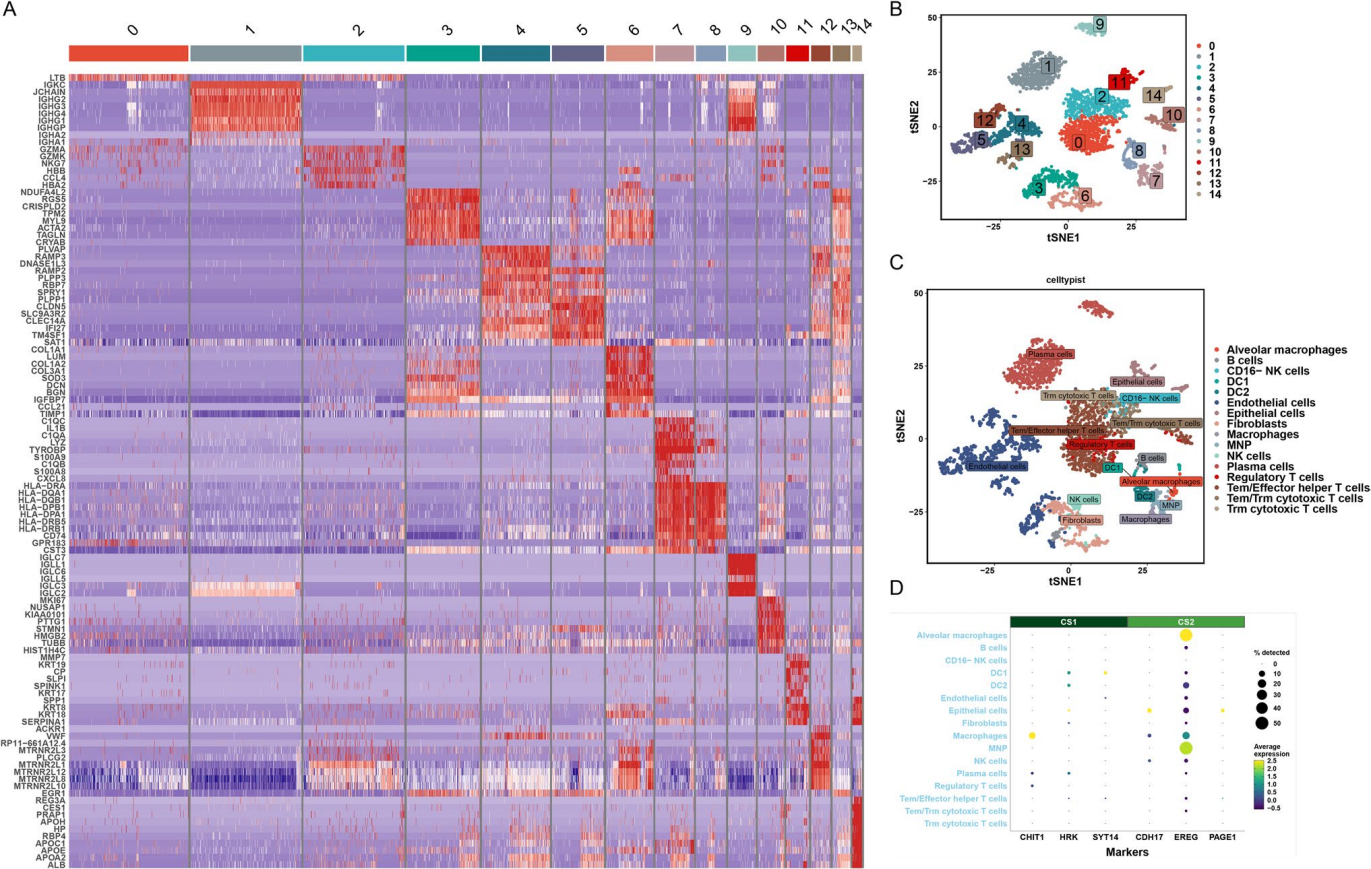
Differential expression of prognosis-related genes in immune cells from the CS1 subtype. **A** Heatmap of the expression level of characteristic genes from each subtype. **B** The cell tSNE clustering result. **C** The result of cellTypist annotation. **D** Bubble plot of gene expression for prognosis-related genes in each type of immune cell

**Fig. 6:**
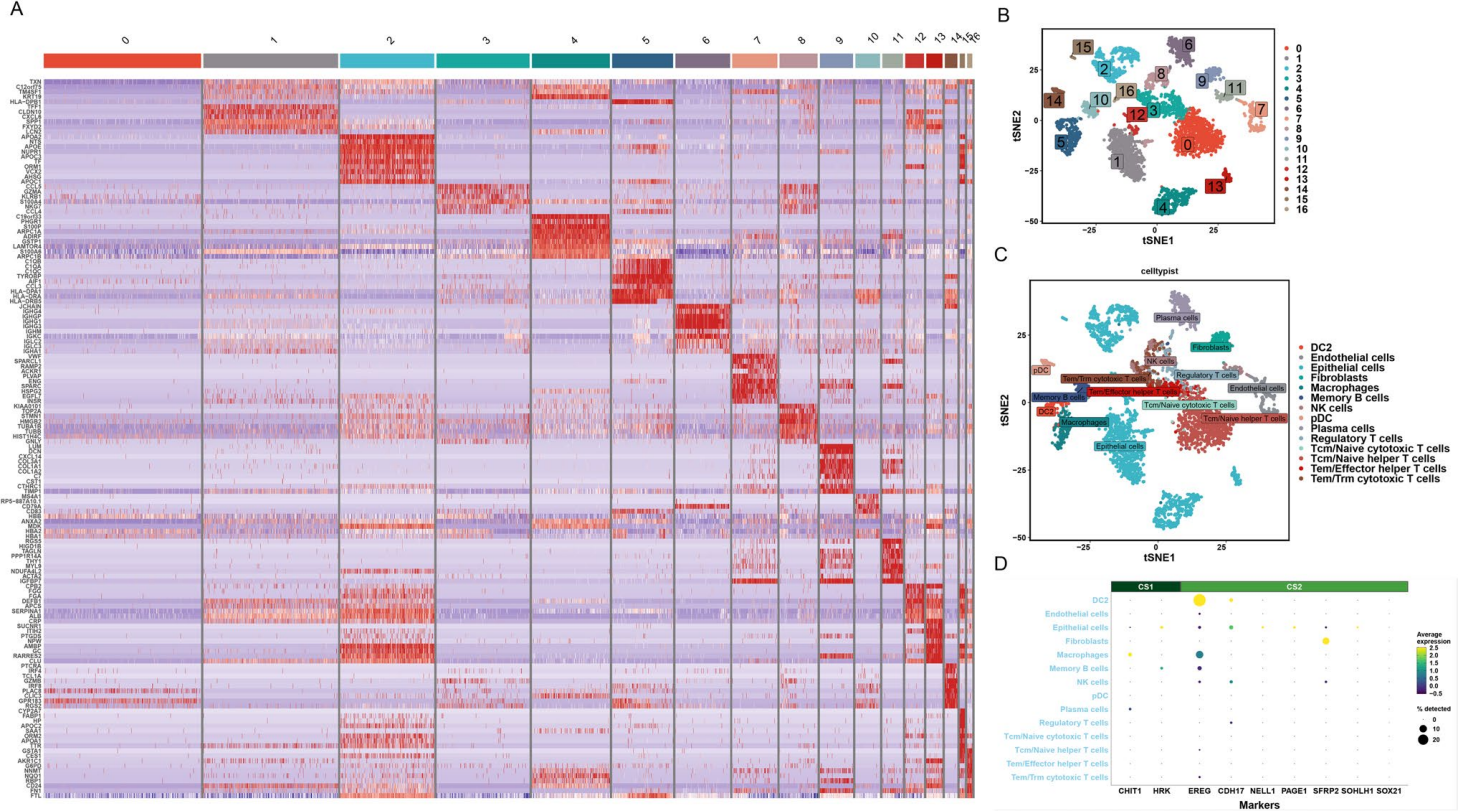
Differential expression of prognosis-related genes in immune cells from the CS2 subtype. **A** Heatmap of the expression levels of characteristic genes from each subtype. **B** The cell tSNE clustering result. **C** The result of cellTypist annotation. **D** Bubble plot of gene expression for prognosis-related genes in each type of immune cell

Comprehensive analysis of the prognostic genes was performed in both subtypes in single-cell datasets. Analysis of the characteristics of the CS1 subtype in single-cell data is shown in Fig. 5, while that of CS2 is shown in Fig. 6. In Figs. 5 and 6, A shows the heatmap for expression levels of the characteristic genes in each subtype, B shows the cell tSNE clustering result, C is the cellTypist annotation, and D shows the bubble plot for prognosis-related gene expression in each immune cell type. The prognosis-related gene EREG was highly expressed in several types of immune cell.

### Comparison of EREG expression and its role in HepG2 and HUH7 cells

**Fig. 7:**
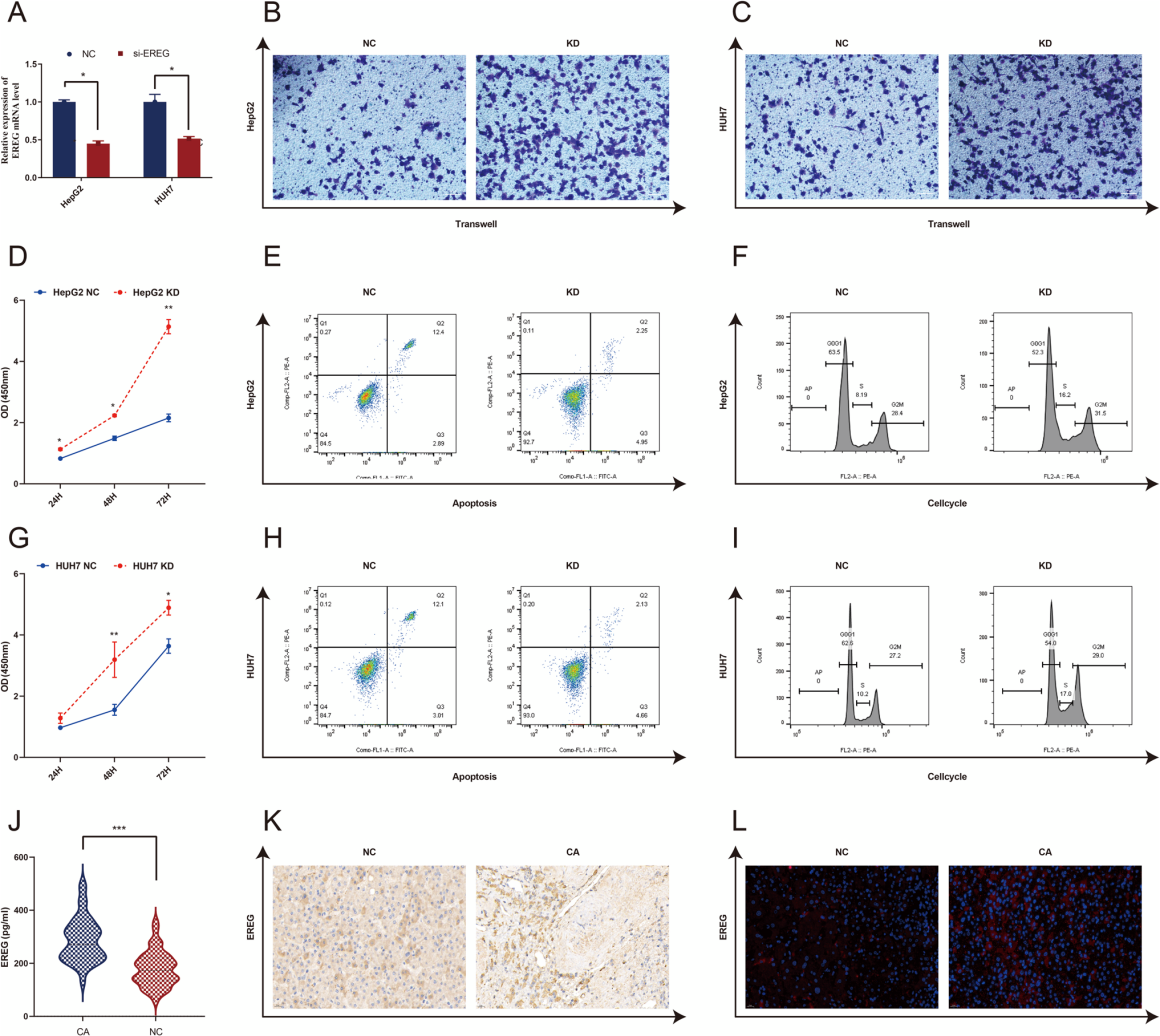
In vivo and in vitro experiments for the validation of EREG. **A** qRT-PCR validation of EREG expression in HepG2 and HUH7 cells after EREG knockdown. **B**, **C** Migration assay results for HepG2 and HUH7 cells in the EREG-siRNA group (KD) and in the CON-siRNA group (NC). Scale bar: 100 μm. **D**, **G** Proliferation ratio of HepG2 and HUH7 cells as determined by CCK-8 assays in the EREG-siRNA group (KD) and in the CON-siRNA group (NC). **E**, **H** Apoptosis detection results after 24 h in HepG2 (**E**) and HUH7 cells (**H**) for the EREG-siRNA (KD) and CON-siRNA groups. **F**, **I** Cell proliferation results after 24 h in HepG2 (**F**) and HUH7 cells (**I**) for the EREG-siRNA (KD) and CON-siRNA groups. Expression of EREG in the serum (**J**) and liver tissue (**K**, **L**) of HCC and healthy control patients

We included 50 cases of HCC patients and 50 cases of healthy controls in CS1 and CS2 and then compared EREG expression in the serum and liver between the two groups. HCC patients showed significantly higher EREG expression than controls. A comparison of the clinical characteristics and EREG expression between CS1 and CS2 is shown in Table 1. We also performed in vitro experiments to investigate the effect of EREG on the HCC cell lines HepG2 and HUH7. Knockdown of the EREG target gene (Fig. 7A) resulted in significant anti-apoptosis, pro-proliferation and pro-migration effects in HepG2 cells (Fig. 7B, D–F) and in HUH7 cells (Fig. 7C, G–I). Moreover, HCC patients had significantly higher EREG levels in serum (Fig. 7J) and liver tissue (Fig. 7K, L) than healthy controls.

**Table 1:** Clinical characteristics of patients

Characteristics	HCC (*n* = 50)	NC (*n* = 50)	*P*
Age (yrs)	65.00 (55.00–69.25)	51.00 (46.00–54.00)	< 0.001
Female (%)	9.00 (18)	11.00 (21)	0.617
White blood cell count (10^9^/L)	4.47 ± 1.77	5.83 ± 1.53	< 0.001
Neutrophil (10^9^/L)	2.55 (1.60–3.63)	3.00 (2.50–3.95)	0.015
Lymphocyte (10^9^/L)	1.00 (0.70–1.40)	1.90 (1.60–2.30)	< 0.001
Haemoglobin (g/L)	123.90 ± 22.30	147.68 ± 14.44	< 0.001
Platelet count (10^9^/L)	110.50 (70.75–150.75)	238.50 (198.00–274.25)	< 0.001
Albumin (g/L)	37.20 ± 5.86	46.89 ± 2.16	< 0.001
Alanine aminotransferase (U/L)	34.50 (22.50–48.25)	23.00 (19.75–27.75)	< 0.001
Aspartate aminotransferase (U/L)	22.50 (17.75–32.25)	24.00 (16.00–34.00)	0.809
Alkaline phosphatase (U/L)	98.00 (71.00–133.25)	72.00 (58.00–83.25)	< 0.001
γ-glutamyl transpeptidase (U/L)	46.50 (25.00–73.35)	20.50 (15.00–32.00)	< 0.001
Total bilirubin (μmol/L)	14.55 (10.43–22.03)	11.80 (8.50–14.55)	0.012
Glucose (mmol/L)	5.15 (4.63–5.66)	5.07 (4.75–5.65)	0.669
Creatinine (μmol/L)	67.05 (55.28–74.45)	69.90 (60.58–76.13)	0.357
Triglyceride (mmol/L)	0.99 (.64–1.27)	1.31 (0.92–2.05)	0.001
Total cholesterol (mmol/L)	5.02 ± 0.88	4.11 ± 1.74	0.010
High-density lipoprotein (mmol/L)	1.13 ± 0.46	1.43 ± 0.33	< 0.001
Low-density lipoprotein (mmol/L)	2.34 (1.66–3.09)	3.01 (2.61–3.48)	0.001
Ferritin (ng/ml)	277.95 (144.90–506.08)	163.00 (87.30–252.90)	0.003
Alpha fetoprotein (ng/ml)	16.3 (2.15–1136.15)	2.55 (1.30–3.43)	< 0.001
**Epiregulin (ng/ml)**	**271.00 (221.01–336.53)**	**172.50 (133.77–220.49)**	< 0.001

Data are presented as the means ± SD, medians with (p25, p75), or percentages (numbers of patients)

## Discussion

LncRNAs are intensively studied in both RNA biology and oncology (Hosono et al. 2017). These can enhance characteristic tumor properties such as abnormal cell proliferation, metabolism and metastasis (Xue et al. 2023), thereby leading to hepatocarcinoma (Liu et al. 2015). Regulation of immune responses by lncRNA is a key factor in determining the liver microenvironment and chronic liver disease (Huang et al. 2020). Moreover, immune gene expression is closely linked to tumor development and progression, with immunotherapy now used in various cancer treatments (Wang et al. 2021). In-depth exploration of the association between lncRNAs and immune genes may therefore lead to novel therapeutic strategies for HCC treatment. Furthermore, our prognostic model based on lncRNA-related immune genes may be used by clinicians to more accurately predict the response to immunotherapy and patient outcome.

We developed a novel lncRNA-derived, immune-related prognostic score based on machine learning integration. This was able to predict 5-year survival rates for HCC patients with an AUC as high as 0.912, which is significantly higher than current prognostic scoring systems (Cai et al. 2021, 2022; Lu et al. 2022; Chen et al. 2022). Cases were separated into high- or low-score groups according to the overall median score. Patients with a high score had significantly better prognosis than those with a low score. Our analyses revealed significant differences between groups for tumor metabolism, microenvironment and gene mutation characteristics. Formation of the tumor microenvironment depends mainly on tumor metabolism, since nutrients in the microenvironment are hijacked to promote malignant progression. Multiple studies have suggested that tumor metabolism, the tumor microenvironment, and gene mutation are tightly linked to cancer development, growth, metastasis and prognosis (Xia et al. 2021; Togo et al. 2020; Bader et al. 2020). Further comparison of responses to ICB therapy, chemotherapy, and targeted therapy revealed that patients with a high score had better response to treatment than those with a low score. This result suggests our prognostic model may also have good accuracy for predicting treatment response in HCC patients. In conclusion, we have demonstrated several ways in which our prognostic risk score model may improve the accuracy of HCC diagnosis and the effectiveness of immunotherapy.

Due to the complexity and diversity of immune cell functions in the tumor microenvironment (TME) (Xue et al. 2021), therapeutic strategies that target only specific elements are often ineffective. Our risk score model shows that the high-score group with better prognosis has a greater density of immune cells, especially T cells, B cells, NK cells and macrophages. These findings provide new insights into the role of infiltrating immune cells in HCC-TME. Hypoxia in hepatocellular carcinoma can induce NK cells to upregulate HIF-1α, which then alters the expression of glycolytic enzymes, metabolite transporters and enzymes involved in biosynthesis, thereby affecting the metabolism of immune cells (Li et al. 2019). In the TME induced by chronic inflammation, the suppression of chronic inflammation by T cells is associated with good prognosis (Shan et al. 2022). In the early stages of HCC tumorigenesis, macrophages (M1 type) participate in the immune clearance of tumor cells. However, in the later stages the M2 macrophage type participate in the metastasis of tumor cells, thereby avoiding the immune system. B cells are antigen-presenting cells that promote cytokine secretion and promote liver cancer metastasis. They can also directly kill tumor cells by secreting granzyme B (Xue et al. 2022^), (^Chen et al. 2023).

Immunotherapy has greatly changed the treatment landscape for HCC, but its efficacy varies significantly between patients. It is therefore critical to find biomarkers that can accurately predict the response to treatment. In view of the complexity of immune cell interactions, development of novel immunotherapies for HCC and identification of accurate biomarkers should be based on the presence of multiple immune cell types (Ruf et al. 2021). Cluster analysis of single cell sequencing data was used here to accurately identify immune cell types. We found that Epiregulin (EREG), a gene with prognostic significance, showed high expressed levels in several immune cell types. Most normal tissues show low levels of EREG expression, but the levels are elevated in different cancer types and promote tumor progression by activating EGFR signaling. EREG plays key roles in regulating physiological stress, inflammation and angiogenesis (Cheng et al. 2021). High levels of EREG expression in colorectal cancer (CRC) associate with better therapeutic outcomes, while EREG overexpression in non-small cell lung cancer may be a therapeutic target for EGFR-TKI (Lin et al. 2020). EREG ^(−/−)^ mice have significantly lower levels of inflammation (macrophages, polynucleated leukocytes, and CXCL1) compared to wildtype mice, as well as significantly reduced tumor incidence (Cheng et al. 2021). Together, these observations suggest EREG has a critical role in cancer development and progression.

The EREG gene has been rarely studied in HCC. In the present work, EREG protein was found to be up-regulated in the sera and liver tissues of HCC patients. However, low EREG expression was reportedly an independent predictor of poor survival and reduced drug sensitivity. In vitro experiments with HepG2 and HUH7 cell lines showed that EREG knockdown mediated by siRNA could increase cell proliferation and migration, accelerate the cell cycle, and inhibit cell apoptosis. EREG has been proposed as a possible biomarker for CRC patients, with high expression linked to better survival of patients who received neoadjuvant chemoradiotherapy (Lin et al. 2020). These findings indicate that upregulation of EREG may inhibit tumor development and improve the effectiveness of immunotherapy in HCC patients.

In summary, we developed a novel prognostic scoring system based on machine learning integration and involving lncRNA-associated immune genes. This model showed excellent accuracy for the prognosis of HCC patients. For HCC patients, it is difficult to achieve satisfactory results using surgery, radiotherapy and chemotherapy. The development of tumors results from the joint action of immune cells and related genes (Fonseca and Araujo 2022). Blocking one of these steps may prevent the formation of metastasis. Further in-depth research of these mechanisms should help to find new immunotherapies that are more effective at treating HCC, either by decreasing tumor promotion or enhancing anti-tumor effects. Furthermore, EREG expression was found to be a biomarker for prognosis and treatment outcome in HCC patients. Moreover, in vitro experiments revealed that EREG could increase cell apoptosis and inhibit the proliferation and migration of cells. EREG is therefore likely to play a major role in the immune response to HCC and could thus be a key target for immunotherapy.

## Supplementary Information

Below is the link to the electronic supplementary material.

Supplementary file1 (XLSX 24 KB)

Supplementary file2 (PDF 367 KB)
