# Supplementary material for: Development of a novel lncRNA-derived immune gene score using machine learning-based ensembles for predicting the survival of HCC
Source: J Cancer Res Clin Oncol. 2024 Feb 9;150(2):86. doi: 10.1007/s00432-024-05608-6 (PMC10858126; doi:10.1007/s00432-024-05608-6)
Supplement: Supplementary file 2 — Supplementary file2 (PDF 367 KB) [file 432_2024_5608_MOESM2_ESM.pdf]

Table S1: The reference of the specific siRNA for human EREG

Targeted gene	Sense（5'-3'）	Antisense（5'-3'）
EREG	CAGGUGUGAAGUGGGUUAUTT	AUAACCCACUUCACACCUGTT

siRNA: Small interfering RNA, EREG: Human epiregulin

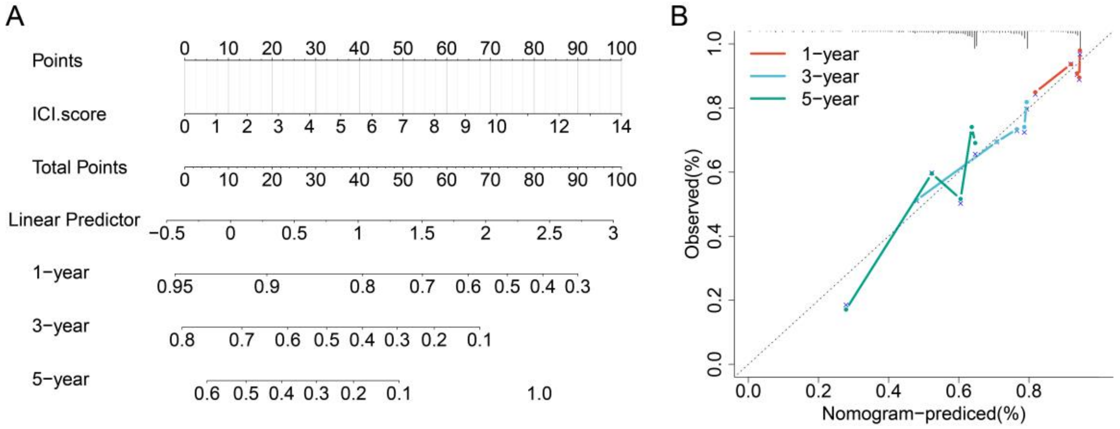

Figure S1: Nomogram used to determine prognostic probabilities in the TCGA-LIHC dataset. (A) Nomogram for predicting the 1-, 3-, and 5-year overall survival (OS) of HCC patients. (B) Calibration curves for 1-, 3-, and 5-year OS of HCC patients.

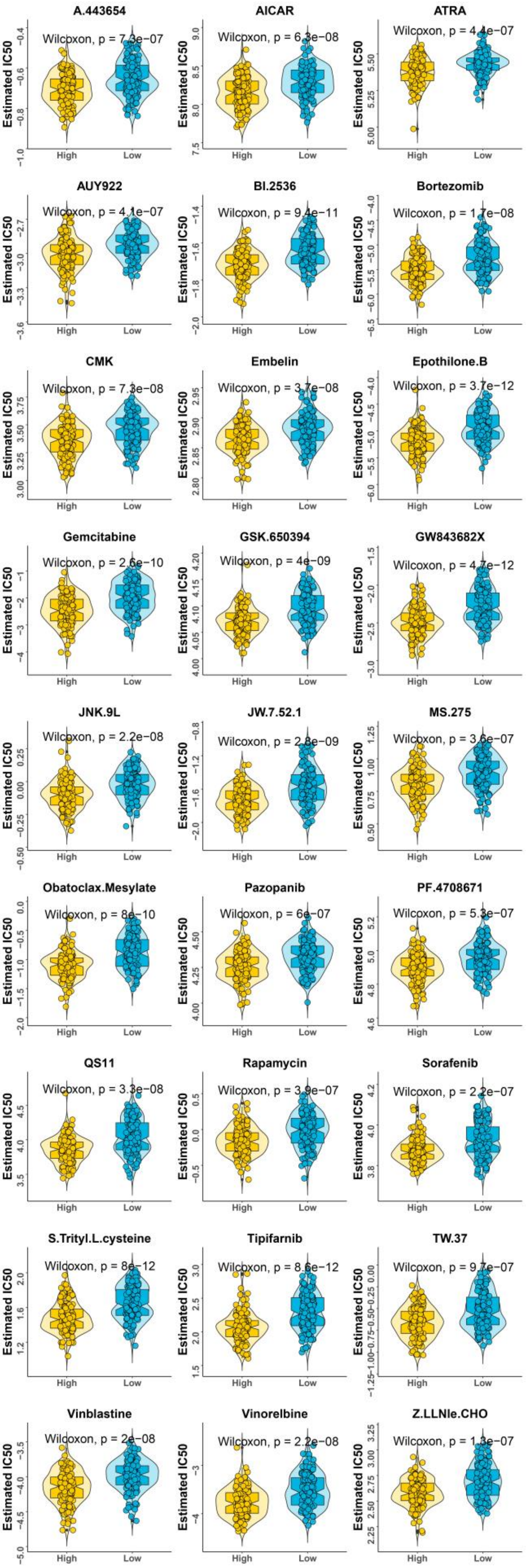
A.443654
Wilcoxon, p = 7.3e−07
AICAR
Wilcoxon, p = 6.3e−08
ATRA
Wilcoxon, p = 4.4e−07
AUY922
Wilcoxon, p = 4.1e−07
BI.2536
Wilcoxon, p = 9.4e−11
Bortezomib
Wilcoxon, p = 1.7e−08
CMK
Wilcoxon, p = 7.3e−08
Embelin
Wilcoxon, p = 3.7e−08
Epothilone.B
Wilcoxon, p = 3.7e−12
Gemcitabine
Wilcoxon, p = 2.6e−10
GSK.650394
Wilcoxon, p = 4e−09
GW843682X
Wilcoxon, p = 4.7e−12
JNK.9L
Wilcoxon, p = 2.2e−08
JW.7.52.1
Wilcoxon, p = 2.8e−09
MS.275
Wilcoxon, p = 3.6e−07
Obatoclax.Mesylate
Wilcoxon, p = 8e−10
Pazopanib
Wilcoxon, p = 6e−07
PF.4708671
Wilcoxon, p = 5.3e−07
QS11
Wilcoxon, p = 3.3e−08
Rapamycin
Wilcoxon, p = 3.9e−07
Sorafenib
Wilcoxon, p = 2.2e−07
S.Trityl.L.cysteine
Wilcoxon, p = 8e−12
Tipifarnib
Wilcoxon, p = 8.6e−12
TW.37
Wilcoxon, p = 9.7e−07
Vinblastine
Wilcoxon, p = 2e−08
Vinorelbine
Wilcoxon, p = 2.2e−08
Z.LLNle.CHO
Wilcoxon, p = 1.3e−07
Estimated IC50
High
Low

Figure S2: Sensitivity to antitumor agents (IC50) in high- and low- score groups. The top 27 drugs with significant differences in sensitivity are shown.
